# Higenamine inhibits IL-1β-induced inflammation in human nucleus pulposus cells

**DOI:** 10.1042/BSR20190857

**Published:** 2019-06-28

**Authors:** Xiaoliang Bai, Wenyuan Ding, Sidong Yang, Xiaohui Guo

**Affiliations:** 1 Department of Spine, the Third Hospital of Hebei Medical University, Shijiazhuang 050051, China; 2 Department of Spine, the Second Hospital of Tangshan City, Tangshan 063000, China

**Keywords:** Intervertebral disc degeneration, higenamine, nucleus pulposus cells (NPCs)

## Abstract

Intervertebral disc degeneration (IDD) is a natural progression of the aging process associated with inflammation. Higenamine, a plant-based alkaloid, has been identified to possess various pharmacological properties, including anti-inflammatory activity. In the present study, we aimed to evaluate the role of higenamine in interleukin (IL)-1β-induced inflammation in human nucleus pulposus cells (NPCs). The results showed that higenamine improved cell viability in IL-1β-induced NPCs. The IL-1β-dependent up-regulation of inflammatory molecules including inducible nitric oxide synthase (iNOS), nitric oxide (NO), prostaglandin E2 (PGE2), cyclooxygenase-2 (COX-2), tumor necrosis factor alpha (TNF-α), and IL-6 was attenuated by higenamine in NPCs. The increased productions of matrix metalloproteinases (MMP-3 and MMP-13), as well as a disintegrin and metalloprotease with thrombospondin motifs (ADAMTS-4 and ADAMTS-5) were significantly mitigated by higenamine treatment. Furthermore, we also found that higenamine suppressed the IL-1β-induced activation of NF-κB signaling pathway in NPCs. In conclusion, the present study proved that higenamine exhibited anti-inflammatory activity against IL-1β-induced inflammation in NPCs via inhibiting NF-κB signaling pathway. These results suggested that higenamine might be a therapeutic agent for the treatment of IDD.

## Introduction

Intervertebral disc degeneration (IDD) is a natural progression of the aging process that common causes lower back pain, representing a serious socio-economic burden [1]. Deeper knowledge of the IDD appears to be important to improve the current therapies. It is evident that IDD is closely associated with inflammatory response, which is mediated by the abnormal production of pro-inflammatory molecules secreted by both the nucleus pulposus (NP) and the annulus fibrosus (AF) [2]. During the degeneration proceeds, an elevation in levels of inflammatory cytokines is observed. Some cytokines such as tumor necrosis factor (TNF)-α, interleukin (IL)-1 α/β, IL-6, and IL-17 can promote matrix degradation, chemokine production, and results in changes in cell phenotype, thereby initiating a cascade of degenerative events [3,4]. In addition to the role in the onset of the inflammatory condition, a number of proinflammatory mediators play crucial role in the development of IDD [3]. Taken together, inhibition of the production of cytokines may be new approach for treating IDD.

Higenamine, a chemical compound found in a variety of plants, has been demonstrated to possess a broad of bioactivities, such as analgesic, anti-tumor, antioxidative, as well anti-inflammatory effects [5–8]. Park et al. [9] reported that higenamine protects a macrophage cell line from LPS-induced inflammation and increases survival rates in LPS-treated mice. Higenamine inhibits inducible nitric oxide synthase (iNOS) expression and reduces nitric oxide (NO) production in LPS-induced macrophages. Yu et al. [6] demonstrated that higenamine protects brain from hypoxia-induced injury. Further investigations reveal that higenamine suppresses expression of high mobility group box-1 (HMGB1), which plays an important role for promoting inflammation in brain ischemia. These findings suggest that higenamine exhibits anti-inflammatory activity. Therefore, we speculated that it might have protective effect on IDD. In the present study, we investigated the effects of higenamine on IL-1β-induced inflammation in human nucleus pulposus cells (NPCs).

## Materials and methods

### NPCs isolation and treatments

The present study was approved by the Ethics Review Board of the Third Hospital of Hebei Medical University (Shijiazhuang, China). Written informed consent was obtained from all participants. Human lumbar disc NP tissues were obtained from symptomatic IDD patients (*n* = 5) underwent microendoscopy discectomy at the Department of orthopedics, the Third Hospital of Hebei Medical University between June 2016 and August 2017. The specimens were isolated within 30 min, and then frozen in liquid nitrogen for the isolation of NPCs.

The NP tissues were washed by ice-cold phosphate-buffered saline (PBS) and then cut into small pieces, followed by digestion in PBS containing 0.025% type II collagenase (Invitrogen, Carlsbad, CA, U.S.A.) for approximately 4 h at 37°C. The digested tissues were filtered and centrifuged at 500 *g* for 10 min. The supernatant was discarded, the cells were suspended in DMEM/F12 medium (Hyclone, Logan, UT, U.S.A.) containing 15% fetal bovine serum (FBS, Hyclone) and seeded into culture dishes. The cells were maintained in an incubator with 5% CO_2_ at 37°C. Cells at passage 1–3 were used for all experiments.

### Cell viability assay

MTT assay was performed to evaluate the cell viability of NPCs. NPCs were seeded in 96-well plates at 5 × 10^3^ cells per well, followed by an incubation with different concentrations of higenamine for 24 h (0, 10, 20, 40, and 80 μM) with or without the presence of IL-1β (10 ng/ml). Then, 20 μl of MTT solution (5 mg/ml) was added to each well and incubated for 4 h at 37°C. Then, each well was added with 150 μl DMSO to dissolve the crystal. Finally, the absorbance at 490 nm was measured using a micro-plate reader (Bio-Rad, Hercules, CA, U.S.A.).

### Measurement of NO

Cell culture supernatants were collected for the detection of NO using Griess method with a total NO and Nitrate/Nitrite Parameter Assay Kit (R&D Systems, Minneapolis, MN, U.S.A.) according to the manufacture’s protocol.

### Quantitative real-time PCR

Total RNA was isolated from chondrocytes using TRIzol (Invitrogen) in accordance with the manufacturer’s protocol. Then the cDNA was synthesized using the PrimeScript-RT reagent kit. The PCR was performed using SsoAdvanced Universal SYBR Green Supermix (Bio-Rad) on a CFX Connect Real-Time PCR Detection System (Bio-Rad). The specific primers for iNOS were sense, 5′-TTTCCAAGACACACTTCACCA-3′, and antisense, 5′-ATCTCCTTTGTTACCGCTTCC-3′; for cyclooxygenase-2 (COX-2) were sense, 5′-GAGAGATGTATCCTCCCACAGTCA-3′, and antisense, 5′-GACCAGGCACCAGACCAAAG-3′; for TNF-α were sense, 5′-CCTCTCTCTAATCAGCCCTCTG-3′, and antisense, 5′-GAGGACCTGGGAGTAGATGAG-3′; for IL-6 were sense, 5′-GGCACTGGCAGAAAACAACC-3′, and antisense, 5′-GCAAGTCTCCTCATTGAATCC-3′; and for β-actin were sense, 5′-AAATCGTGCGTGACATCAAAGA-3′, and antisense, 5′-GGCCATCTCCTGCTCGAA-3′. The results were calculated using the 2^−ΔΔCT^ method.

### Western blot

Cells from each group were homogenized in radioimmunoprecipitation assay (RIPA) buffer (Beyotime Technology, Shanghai, China) containing 1% protease inhibitor cocktail (Roche, Basel, Switzerland) and phosphatase inhibitor cocktail (Roche). Then the homogenates were centrifuged at 12000 rpm for 15 min at 4°C. Afterward, the supernatants were collected for the detection of protein concentration using the bicinchoninic acid (BCA) protein assay kit (Beyotime). A total of 40 μg proteins from each group were separated on 12% Bis–Tris sodium dodecyl sulfate-polyacrylamide gel electrophoresis gel (SDS-PAGE), and then transferred to a polyvinylidene fluoride (PVDF) membrane (0.45 μm; Millipore, Boston, MA, U.S.A.). The membranes were blocked with 5% nonfat milk for 1 h at room temperature and subsequently immunoblotted with primary antibodies against iNOS, COX-2, TNF-α, IL-6, matrix metalloproteinase (MMP)-3, MMP-13, a disintegrin and metalloprotease with thrombospondin motifs (ADAMTS)-4, and ADAMTS-5, p65, p-p65, IκBα, and β-actin (diluted 1:500; Abcam, Cambridge, MA, U.S.A.) overnight at 4°C. After washing in TBST for three times, the membranes were incubated with HRP-conjugated goat anti-rabbit secondary antibody (diluted 1:5000; Abcam) for 1.5 h at room temperature. After washing with TBST for three times, membranes were stained with chemiluminescent HRP substrate (Millipore). The gray values of the bands were analyzed Image J software (National Institutes of Health, NIH, Bethesda, MD, U.S.A.). GAPDH was used as the control protein.

### ELISA assay

The productions of prostaglandin E2 (PGE2), TNF-α, IL-6, MMP-3, MMP-13, ADAMTS-4, and ADAMTS-5 in the cell culture were tested using corresponding ELISA kits (R&D Systems) according to the manufacturer’s instructions.

### Statistical analysis

The results were presented as means ± S.D. Statistical analyses were performed using Graph-Pad Prism 6.0 (GraphPad Software, Inc, San Diego, CA, U.S.A.). Differences among the different groups were analyzed by one-way analysis of variance (ANOVA) followed by the Tukey’s test. *P* values less than 0.05 were considered significant.

## Results

### Higenamine improved cell viability in NPCs exposed to IL-1β

**Figure 1:**
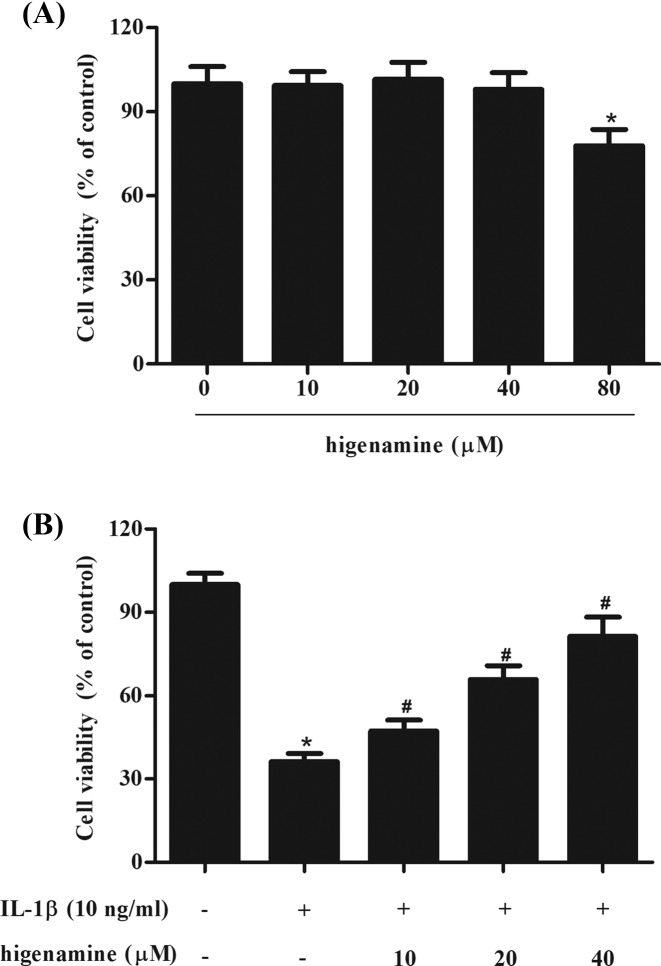
Effect of higenamine on cell viability in NPCs exposed to IL-1β (**A**) NPCs were incubated with different concentrations of higenamine (0, 10, 20, 40, and 80 μM) for 24 h. MTT assay was performed to evaluate cell viability. (**B**) NPCs were pretreated with 10, 20, 40 μM of higenamine for 2 h, followed by the induction of IL-1β (10 ng/ml) for 24 h. Cell viability was detected using MTT assay. *P < 0.05 vs. control NPCs. ^#^*P* < 0.05 vs. IL-1β-stimulated NPCs.

To evaluate the effect of higenamine on the cell viability of NPCs, the cells were incubated with different concentrations of higenamine (0, 10, 20, 40, and 80 μM) for 24 h. MTT assay showed that higenamine did not significantly affect the viability of NPCs up to 40 μM (Figure 1A). The concentrations of 10, 20, 40 μM were selected for the further investigations. Then we evaluated the effects of higenamine on IL-1β-induced NPCs. As shown in Figure 1B, cell viability of NPCs was markedly decreased after IL-1β treatment, while pretreatment with higenamine improved the cell viability.

### Higenamine decreases the production of NO and PGE2 in IL-1β-stimulated NPCs

**Figure 2:**
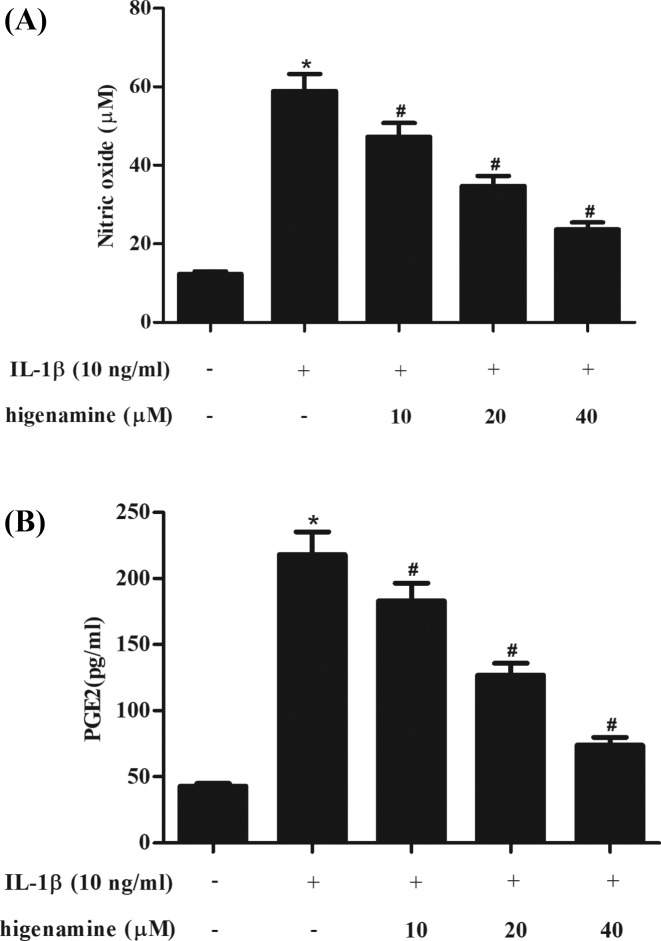
Effect of higenamine on the production of NO and PGE_2_ in IL-1β-stimulated NPCs NPCs were pretreated with 10, 20, 40 μM of higenamine for 2 h, followed by the induction of IL-1β (10 ng/ml) for 24 h. The productions of NO (**A**) and PGE_2_ (**B**) in cell culture were determined. *P < 0.05 vs. control NPCs. ^#^*P* < 0.05 vs. IL-1β-stimulated NPCs.

Then we assessed the effect of higenamine on IL-1β-induced production of NO and PGE_2_ in NPCs. Results in Figure 2A,B showed that the production of NO and PGE_2_ was obviously increased in IL-1β-stimulated NPCs. Pretreatment with higenamine attenuated the induction of NO and PGE_2_ production in NPCs.

### Higenamine inhibits the expressions of iNOS and COX-2 in IL-1β-stimulated NPCs

**Figure 3:**
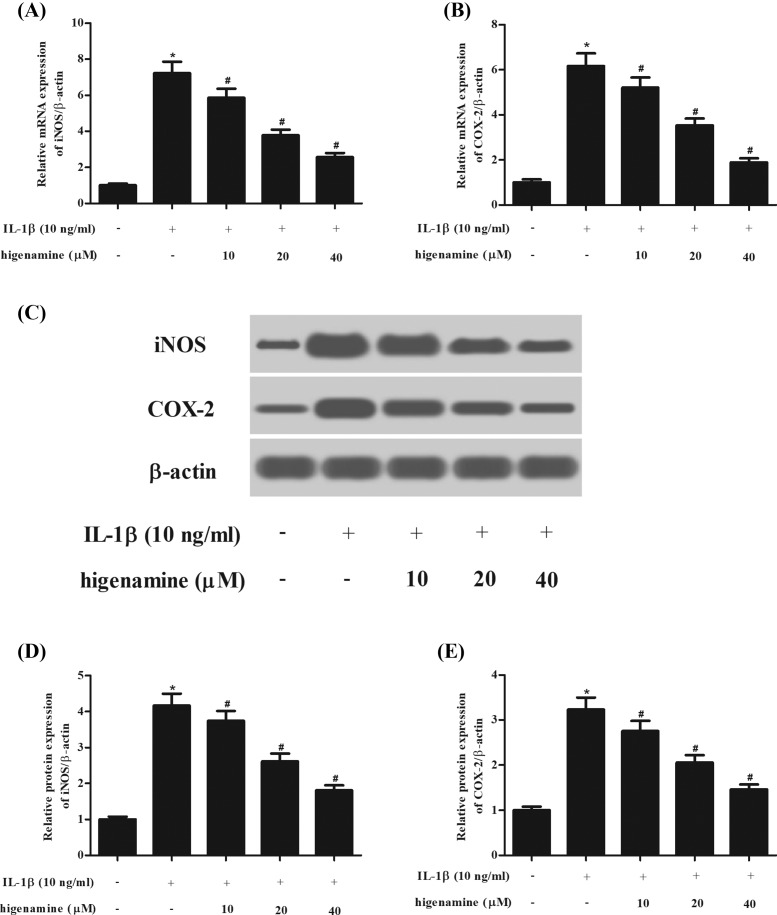
Effect of higenamine on the expressions of iNOS and COX-2 in IL-1β-stimulated NPCs NPCs were pretreated with 10, 20, 40 μM of higenamine for 2 h and then treated with IL-1β (10 ng/ml) for 24 h. (**A,B**) The mRNA expression levels of iNOS and COX-2 were determined using quantitative real-time PCR (qRT-PCR). (**C**) The expressions of two inflammatory mediators, iNOS and COX-2, were measured using Western blot analysis. (**D,E**) Quantification analysis of iNOS and COX-2. *P < 0.05 vs. control NPCs. ^#^*P* < 0.05 vs. IL-1β-stimulated NPCs.

Next, the expressions of two inflammatory mediators, iNOS and COX-2, were measured using Western blot analysis. As indicated in Figure 3, IL-1β stimulated both mRNA and protein expression levels of iNOS and COX-2 in NPCs. However, the induction of iNOS and COX-2 was mitigated by higenamine pretreatment.

### Higenamine suppresses the levels of TNF-α and IL-6 in IL-1β-stimulated NPCs

**Figure 4:**
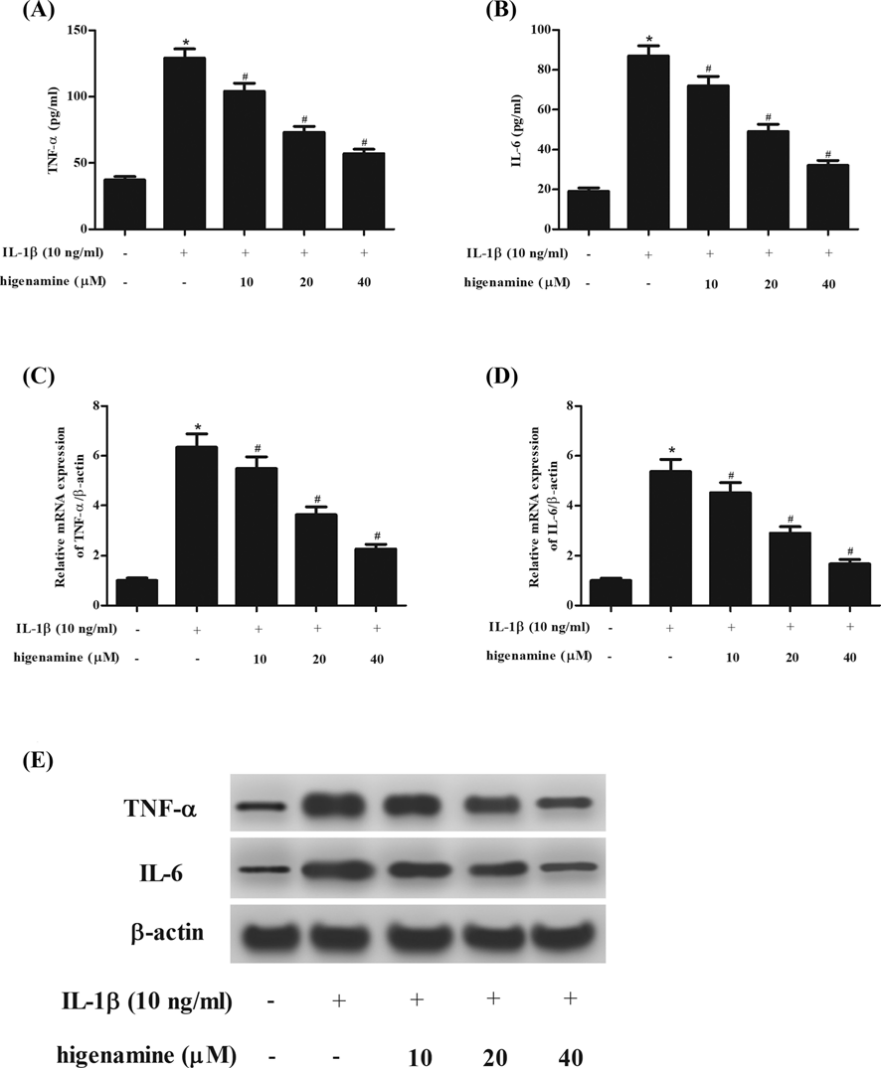
Effect of higenamine on the secretion of TNF-α and IL-6 in IL-1β-stimulated NPCs NPCs were stimulated with IL-1β (10 ng/ml) for 24 h in the presence or absence of higenamine (10, 20, 40 μM). The production of TNF-α (**A**) and IL-6 (**B**) in cell culture was determined using ELISA. (**C,D**) The mRNA expression levels of TNF-α and IL-6 were determined using qRT-PCR. (**E**) The protein expressions of TNF-α and IL-6 were measured using Western blot. *P < 0.05 vs. control NPCs. ^#^*P* < 0.05 vs. IL-1β-stimulated NPCs.

In order to investigate the effect of higenamine on inflammatory cytokines, the production of TNF-α and IL-6 was determined using ELISA. The results proved that IL-1β stimulation caused significant increase in the production of TNF-α and IL-6, while the increased production of TNF-α and IL-6 were suppressed by higenamine (Figure 4A,B). Furthermore, higenamine significantly suppressed the mRNA and protein expression levels of TNF-α and IL-6 induced by IL-1β in NPCs (Figure 4C–E).

### Higenamine inhibits IL-1β-induced production of MMPs and ADAMTSs in NPCs

**Figure 5:**
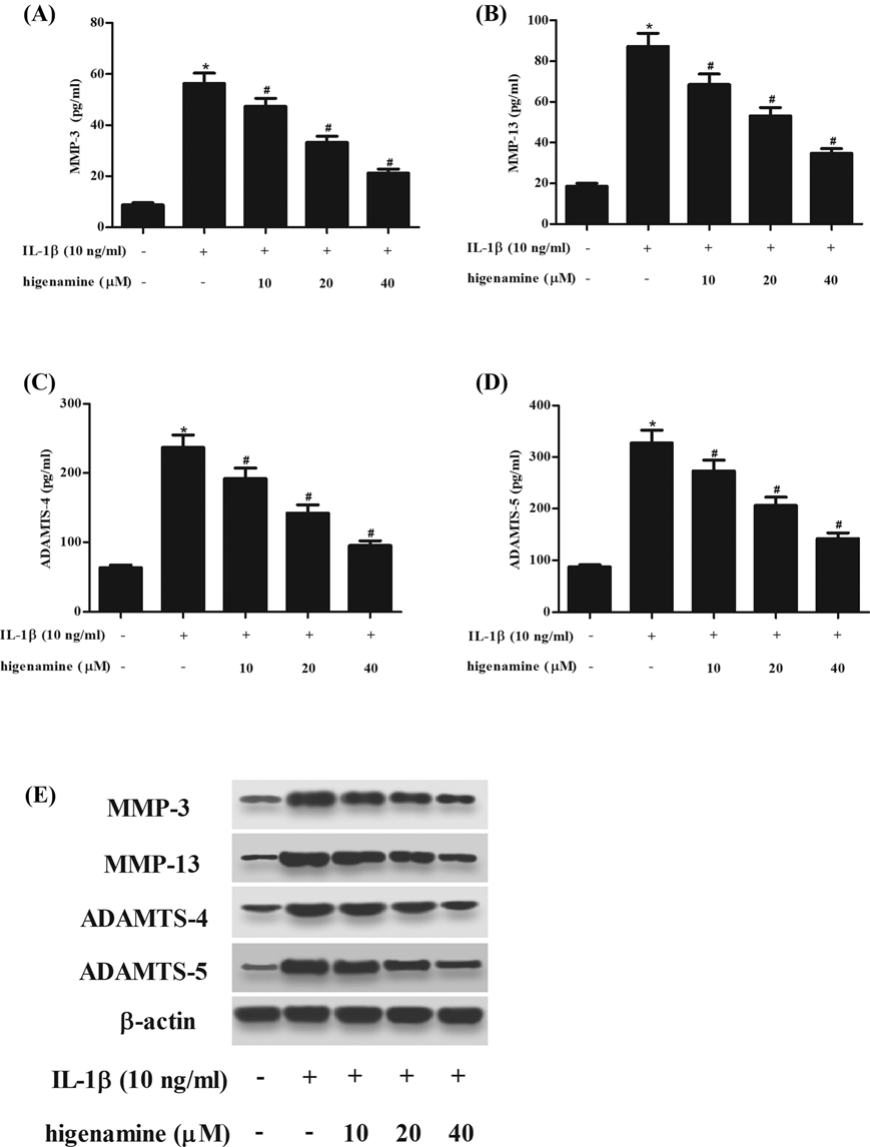
Effect of higenamine on the production of MMPs and ADAMTSs in IL-1β-induced NPCs NPCs were stimulated with IL-1β (10 ng/ml) for 24 h with or without the pretreatment of higenamine (10, 20, 40 μM) for 2 h. The production of matrix degrading enzymes including MMP-3 (**A**), MMP-13 (**B**), ADAMTS-4 (**C**), and ADAMTS-5 (**D**) in cell culture was determined using ELISA. (**E**) The protein expressions of MMP-3, MMP-13, ADAMTS-4, and ADAMTS-5 were determined using Western blot. *P < 0.05 vs. control NPCs. ^#^*P* < 0.05 vs. IL-1β-stimulated NPCs.

Then we explored the effect of higenamine on the production of matrix degrading enzymes including MMP-3, MMP-13, ADAMTS-4, and ADAMTS-5. ELISA results demonstrated that IL-1β-induced production of MMPs and ADAMTSs in NPCs were significantly mitigated by higenamine (Figure 5A–D). In addition, higenamine significantly suppressed the protein expression levels of MMP-3, MMP-13, ADAMTS-4, and ADAMTS-5 induced by IL-1β in NPCs (Figure 5E).

### Higenamine inhibits the activation of NF-κB pathway in IL-1β-stimulated NPCs

**Figure 6:**
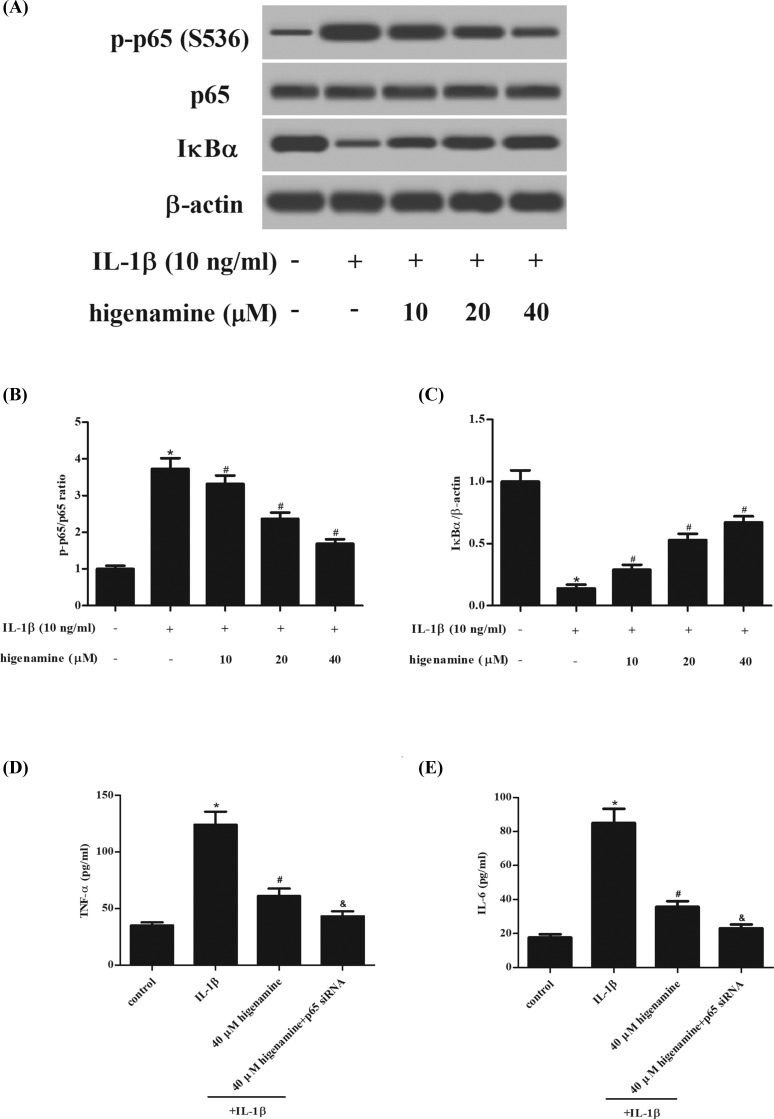
Effect of higenamine on IL-1β-stimulated activation of NF-κB pathway in NPCs (**A**) The expressions of IκBα, NF-κB p65 and p-p65 were measured using Western blot after stimulation with higenamine (10, 20, 40 μM) in the presence or absence of IL-1β (10 ng/ml) for 1 h. (**B,C**) Quantification analysis of p-p65/p65 and IκBα/β-actin. (**D,E**) NF-κB-p65 siRNA-enhanced anti-inflammatory actions of higenamine in IL-1β-stimulated NPCs. *P < 0.05 vs. control NPCs. ^#^*P* < 0.05 vs. IL-1β-stimulated NPCs. &*P* < 0.05 vs. IL-1β+ higenamine.

It is well known that NF-κB pathway plays a crucial role in the inflammatory response [10]. In order to test whether NF-κB pathway was involved in the anti-inflammatory effect of higenamine, the expressions of IκBα, NF-κB p65 and p-p65 were measured using western blot. The results showed that IL-1β treatment greatly induced p-p65 expression and IκBα degradation in NPCs; while, these effects were attenuated by higenamine, indicating that higenamine suppressed the activation of NF-κB pathway (Figure 6A–C). Furthermore, we evaluated the effect of NF-κB-p65 siRNA on the higenamine-mediated effect in IL-1β-stimulated NPCs. As expected, NF-κB-p65 siRNA significantly enhanced the effects anti-inflammatory actions of higenamine in NPCs (Figure 6D,E).

## Discussion

A healthy disc requires a balanced homeostatic environment, however, when the microenvironment is disrupted, a catabolic cascade of events occurs, resulting in up-regulation of proinflammatory cytokines [2]. A variety of inflammatory mediators have been observed to be implicated in the degeneration of the intervertebral disc, such as NO, interleukins, MMPs, PGE2, TNF-α, and a group of cytokines. These mediators result in increased degradative enzymes and a loss of matrix proteins, contributing to the degenerative process of the intervertebral disc [3]. The central NP region is most severely affected during the degeneration of disc degeneration, consequently, it is a focus for novel cell-based regenerative strategy [11]. In the present study, we used IL-1β to stimulate inflammatory response in NPCs to simulate IDD *in vitro*. IL-1β stimulation caused significant decrease in cell viability of NPCs.

Higenamine is a plant-based alkaloid initially isolated from Aconitum and has been identified as the active cardiotonic component of Aconitum. A large number of studies have uncovered various pharmacological properties and its potential therapeutic effects for many diseases including heart failure, disseminated intravascular coagulation (DIC), ischemia/reperfusion (I/R) injury, arthritis, asthma, and erectile dysfunction [8]. In this study, we found that 80 μM higenamine significantly affect the viability of NPCs, perhaps the main reason is that 80 μM higenamine destroyed the structure of NPCs. In addition, pretreatment with higenamine improved the cell viability of IL-1β-induced NPCs, indicating that higenamine might exert anti-inflammatory activity in IL-1β-induced NPCs.

NOSs are a family of enzymes that catalyze the production of NO, which is an important cellular signaling molecule [12]. The inducible isoform, iNOS, produce large amounts of NO and has been found to be involved in inflammatory response [13]. PGE2 is a bioactive lipid that elicits a wide range of biological effects associated with inflammation [14]. COX-2 is an enzyme that is unexpressed under normal conditions, while the expression is elevated, along with the up-regulation of PGEs during inflammation. Previous studies have demonstrated that iNOS, PGE2, NO, and COX-2 are critical inflammatory mediators in the pathogenesis of IDD [3]. In line with the results of previous, herein, we showed that IL-1β stimulated the production of iNOS, PGE2, NO, and COX-2 in NPCs. Besides, two major inflammatory cytokines, TNF-α and IL-6, were also induced by IL-1β in NPCs. Higenamine suppressed the IL-1β-induced production of these inflammatory mediators and cytokines in NPCs.

In healthy discs, there is a balance between synthesis and breakdown of the ECM. During the progress of IDD, the ECM breakdown prevails over its synthesis [15,16]. Degradation of the ECM components results in a number of severe consequences, such as dehydration and fibrosis of the NP, disorganization of the AF, and calcification of the cartilaginous end plates, which may lead to further impairment of Intervertebral disc function [17]. The major components of ECM within the IDD are type II collagen (Col II) and aggrecan. MMPs are a very large family of calcium-dependent, zinc-containing endopeptidases. ADAMTSs are a newly discovered type of metalloproteinase family. MMPs and ADAMTSs are primary enzymes that degrade collagens and aggrecan [18]. Growing body of evidence indicates that many members of MMPs and ADAMTSs are highly expressed in degenerative intervertebral disc tissue and cells, and closely associated with ECM breakdown and the process of disc degeneration [18,19]. Our results showed that IL-1β induced production of MMP-3, MMP-13, ADAMTS-4, and ADAMTS-5 in NPCs. The increased production of MMPs and ADAMTSs were significantly mitigated by higenamine treatment.

NF-κB is an important signaling that plays a crucial role in the regulation of cytokine production and cell survival in inflammation condition [20–22]. IKK complex comprises the kinases IKK1, IKK2 and the scaffold protein IKKγ/NEMO. IKK1 and IKK2 catalyze the cytoplasmic liberation and nuclear translocation of various NF-κB subunits. In response to a number of external stimuli, the complex is activated, resulting in the phosphorylation and subsequent proteasome-mediated degradation of IκB proteins, which activates the transcription of genes that regulate many biological processes, including inflammation [23,24]. Incorrect activation of NF-κB signaling pathway has been found to be linked to diverse diseases, such as cancers, inflammatory, and autoimmune diseases [10,25]. Previous study reported that IL-1β regulates the expressions of Col II and aggrecan through regulating asporin expression via the NF-κB p65 pathway in NPCs during IDD [26]. Sun et al. [27] demonstrated that BAY11-7082, specific inhibitor of NF-κB, significantly inhibits IL-1β-induced NF-κB activation. IL-1β-dependent gene up-regulation of MMP-3, MMP-9, MMP-13, ADAMTS-4, ADAMTS-5 is significantly reduced by BAY11-7082. Besides, the decreased expression of aggrecan and Col II caused by IL-1β is also reversed by BAY11-7082. These findings suggest that the NF-κB signaling pathway is an important mediator of IDD and may be a therapeutic target for alleviating the IDD. In the present, we demonstrated that the IL-1β-induced activation of NF-κB signaling pathway was suppressed by higenamine. Runx protein family comprises three transcription factors Runx1, Runx2, and Runx3 and is closely related with NF-κB [28]. Sato et al. reported that both in the mouse model of IVD degeneration and in human patients with IVD degeneration, there was significant up-regulation of Runx2 expression [29]. However, whether higenamine affects Runx activity in NPCs will require further experiments.

In conclusion, our study proved that higenamine improved cell viability and suppressed inflammation in IL-1β-induced NPCs via inhibiting the activation of NF-κB signaling pathway. The results suggested that higenamine might be a therapeutic agent for the treatment of IDD.

## Abbreviations

ADAMTS: a disintegrin and metalloprotease with thrombospondin motifs
AF: annulus fibrosus
COX-2: cyclooxygenase-2
IDD: intervertebral disc degeneration
IL: interleukin
iNOS: inducible nitric oxide synthase
MMP: matrix metalloproteinase
NO: nitric oxide
NP: nucleus pulposus
NPC: nucleus pulposus cell
PBS: phosphate-buffered saline
PGE2: prostaglandin E2
TNF: tumor necrosis factor
